# Topical Interferon Alpha 2b in the Treatment of Refractory Diabetic Macular Edema

**DOI:** 10.18502/jovr.v15i4.7785

**Published:** 2020-10-25

**Authors:** Arash Maleki, Andrew Phillips Stephenson, Fedra Hajizadeh

**Affiliations:** ^1^Massachusetts Eye Research and Surgery Institution, Waltham, MA, USA; ^2^Ocular Immunology and Uveitis Foundation, Waltham, MA, USA; ^3^Noor Ophthalmology Research Center, Noor Eye Hospital, Tehran, Iran; ^4^University of Cincinnati College of Medicine, Cincinnati, OH, USA

**Keywords:** Diabetic Macular Edema, Interferon α2b

## Abstract

**Purpose:**

To report the efficacy of topical interferon alpha 2b in the treatment of refractory diabetic macular edema.

**Methods:**

In this retrospective interventional case series, five eyes of three individuals with diabetic macular edema resistant to multiple intravitreal injections of anti-vascular endothelial growth factor drugs and macular photocoagulation were included.

**Results:**

All studied eyes had undergone multiple intravitreal injections including bevacizumab, combination of bevacizumab and triamcinolone and aflibercept, and macular laser photocoagulation before being included in this study. Two intravitreal ranibizumab injections had also been performed in both eyes of one patient. Two eyes had undergone pars plana vitrectomy, one for diabetic macular edema and the other for rhegmatogenous retinal detachment. After a discussion regarding the experimental topical interferon alpha 2b treatment, all patients agreed to start interferon alpha 2b drops four times a day. One month after the treatment, optical coherence tomography demonstrated a significant improvement in macular structure and thickness which was stable or improved at the three-month follow-up visit. Visual acuity in all eyes was stable or improved throughout the three-month follow-up period. Conjunctival injection and follicular conjunctivitis were the side effects of topical interferon alpha 2b and were treated with lubrication and steroids.

**Conclusion:**

This case series demonstrated the potential efficacy of interferon alpha 2b in the treatment of refractory diabetic macular edema. It might be an option in patients with contraindications for intravitreal injections.

##  INTRODUCTION

Diabetic macular edema (DME) is one of the most common causes of visual loss in the working-age population and can occur at any stage of diabetic retinopathy.^[1]^ The prevalence of DME in patients with diabetic retinopathy is 2.7–11%, depending on the type and duration of diabetes.^[2,3,4,5,6]^

The development of intraocular anti-vascular endothelial growth factor (anti-VEGF) drugs has revolutionized the treatment of DME in recent years and has widely replaced macular laser photocoagulation (MPC); however, the response to anti-VEGF therapy can be incomplete in some patients with DME despite multiple injections.^[7]^

Both animal and human clinical studies have demonstrated the importance of inflammatory processes in the pathogenesis of DME. Various cytokines and chemokines, including IL-6, IL-8, tumor necrosis factor-alpha (TNF-α), nuclear factor kappa-light-chain-enhancer of activated B cells, protein kinase C, monocyte chemotactic protein, and nitric oxide synthase in addition to VEGF are all key components to this pathogenesis.^[8,9]^

Interferons, a large group of glycoproteins, act against VEGF and other cytokines such as IL-8, IL-10, tissue growth factor beta (TGF-β), and TNF-α through inhibiting their production.^[10]^ Moreover, they enhance the barrier function of retinal microvasculature leading to a more unassailable retinal structure.^[11]^ Interferon alpha has an important role in the treatment of various types of vision-threatening uveitis.^[12]^ Recently, topical interferon alpha 2b (INF-α2b) has been successfully employed in the treatment of refractory pseudophakic macular edema.^[13]^ Moreover, sub-tenon injection of interferon alpha 2a (INF-α2a) has been effectively utilized in DME.^[14]^

In this case series, topical INF-α2b was investigated for the treatment of refractory DME.

##  METHODS

This study was a retrospective interventional case series. All eyes were resistant to multiple intravitreal anti-VEGF injections and MPC. The experimental topical INF-α2b treatment was discussed with the patients and a written informed consent was obtained. Our compounding pharmacy prepared the topical drops by adding two milliliter of distilled water to one milliliter of INF-α2b in a three MIU vials (3 MIU/ml) (PDferon 3 MIU, Pooyesh Pharmaceuticals, Tehran, Iran). Treatment of topical INF-α2b began with the initial regimen of one drop four times per day for the first two or three months based on the investigators' clinical judgment, including patient's symptoms, subjective visual improvement, best-corrected visual acuity (BCVA) improvement, ocular surface health, patient's tolerability of the regimen and the improvement in retinal structure and contour compared to the baseline evaluation and optical coherence tomography (OCT) findings at the one-month follow-up visit. The drops were then tapered by one drop every two or three months thereafter (Table 1).

##  RESULTS

<statement> <title>Case 1</title>

</statement>

**Figure 1:**
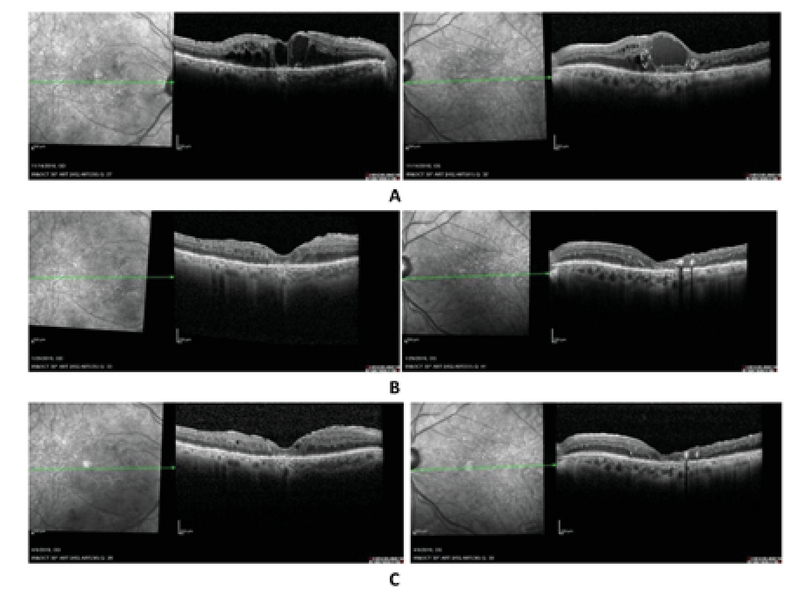
**(A)** Shows the optical coherence tomography (OCT) raster horizontal line passing through the center of the macula in right (upper left) and left eye (upper right) before starting topical interferon alpha 2b treatment. **(B)** and **(C)** are OCTs at one- and three-month follow-up visits, respectively, in the right (left picture) and left (right picture) eyes. In this case, tapering of the drop was started at eight weeks.

A 70-year-old male with a history of type 2 diabetes and hypertension for 16 years presented with severe non-proliferative diabetic retinopathy (NPDR) and DME in November 2007. During his follow-up visits from 2007 to 2018, he received multiple intravitreal injections including bevacizumab, combination of bevacizumab and dexamethasone, combination of bevacizumab and triamcinolone, ranibizumab, and aflibercept in both of his eyes. Additionally, MPC was employed in both eyes and pars plana vitrectomy was used to treat the DME in his left eye. All of these treatment modalities failed (Figure 1A) and his vision decreased to 20/100 and 20/200 from 20/40 and 20/20 in his right and left eye, respectively. After discussing possible treatment options, he agreed to start a topical INF-α2b treatment. During the one-month follow-up visit, his vision improved by one line (to reach 20/80 and 20/100 in the right and left eye, respectively) and OCT showed a significant improvement in macular thickness (Figure 1B). During the three-month follow-up visit, both the visual acuity and the OCT findings were stable (Figure 1C). The patient developed follicular conjunctivitis one and a half months after the treatment initiation with the side effects responding to lubricants and low potency steroids. Tapering of the study medication began at eight weeks.

**Figure 2:**
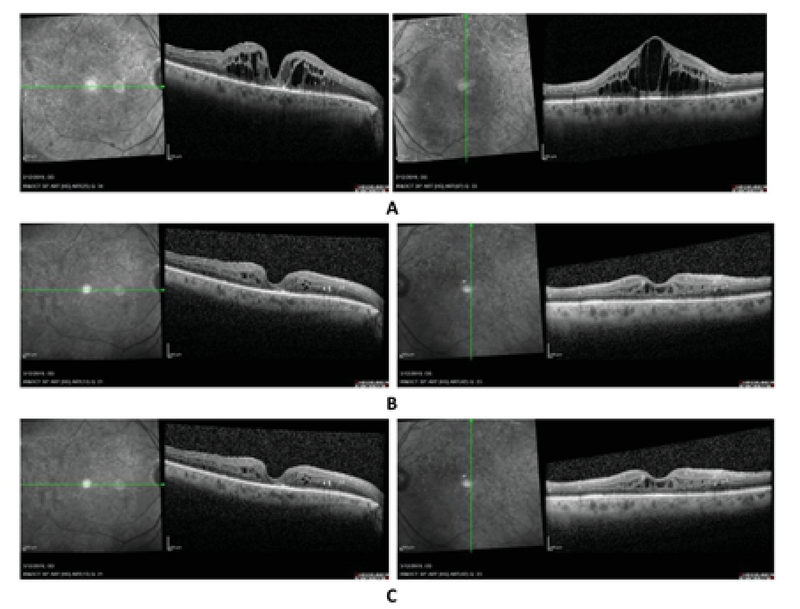
**(A)** Demonstrates the optical coherence tomography (OCT) before initiating the topical interferon alpha 2b treatment. The upper left and upper right are the horizontal raster of the right eye and vertical raster of the left eye, respectively, following the center of the fovea. **(B)** and **(C)** show the OCT at one- and three-month follow-up visits. In this case, tapering of the drop was started at eight weeks.

**Figure 3:**
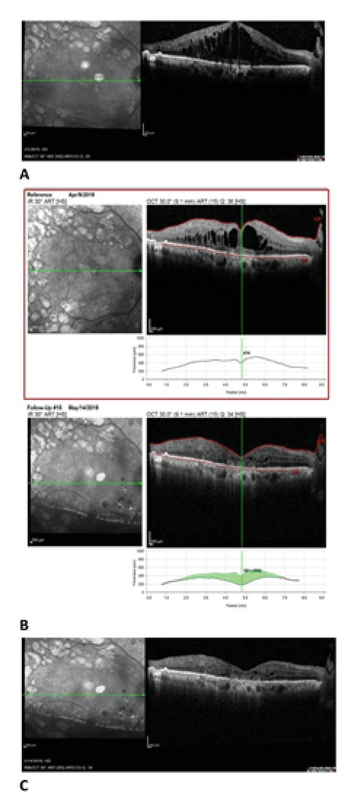
**(A) **The optical coherence tomography (OCT) depicts a significant diabetic macular edema before starting the topical interferon alpha 2b in the right eye. **(B)** The upper picture is the OCT at one-month follow-up visit and indicates a significant improvement; however, this improvement is suboptimal. The lower OCT picture shows the acceptable response one month later (two months after the treatment). The diagram shows the thickness changes between the one- and two-month follow-up visits. **(C) **Shows the stability of the OCT at three-month follow-up visit. In this case, tapering of the drop was started at 12 weeks.

**Table 1:** Treatment protocol


**Treatment protocol**	**Duration of the treatment**
Four times a day	Eight to twelve weeks based on the clinical judgment and the optical coherence tomography findings at one-month follow-up visit.
Three times a day	Eight weeks
Two times a day	Eight weeks
One time a day	Eight weeks
One time every other day	Eight weeks
	
	

<statement> <title>Case 2</title>

</statement>

A 71-year-old male with a history of type 2 diabetes for 13 years, severe NPDR, and DME in his both eyes was presented to us in February 2013. He had undergone cataract surgery in both eyes before presentation and visual acuity was measured at 20/40 in both eyes. For the treatment of DME, he received intravitreal bevacizumab, combination of bevacizumab and triamcinolone, and aflibercept injections in his both eyes. MPC was also utilized in both eyes. During his follow-up visits, he developed rhegmatogenous retinal detachment (RRD) in his left eye and underwent pars plana vitrectomy and silicone oil injection. The silicone oil was removed three months after the primary operation. This individual later developed proliferative diabetic retinopathy (PDR), and panretinal photocoagulation (PRP) was implemented for the treatment of PDR in both eyes. As DME was unresponsive to the aforementioned treatment modalities and other options such as ranibizumab and brolucizumab were not available due to his financial concerns, he was started on topical INF-α2b after discussing the risks and benefits of the treatment. Before treatment initiation, his visual acuity was 20/100 and 20/80 in the right and left eyes, respectively. Figure 2A demonstrates the OCT findings before starting the treatment. Figures 2B and 2C show the changes in the OCT during the one- and three-month follow-up visits, respectively. At the three-month visit, the visual acuity was 20/100 and 20/60 in the right and left eye, respectively. We started to taper the medication at eight weeks.

<statement> <title>Case 3</title>

</statement>

A 64-year-old female with type 2 diabetes and hypertension for 18 years was presented to our clinic. At presentation, in June 2010, she was diagnosed with high-risk PDR and DME in both eyes. She was treated with PRP. She also received multiple intravitreal bevacizumab, combination of bevacizumab and triamcinolone, and aflibercept injections. While the PDR regressed with PRP, the DME in her right eye was resistant to all aforementioned injections. MPC was also performed and was unsuccessful in treating the DME in this eye (Figure 3A). At this point, her vision was 20/100 in the right eye. She agreed to start topical INF-α2b after discussing all treatment options. During her one-month follow-up visit, impressive improvement was observed in the OCT findings (Figure 3B). The visual acuity in the right eye improved by one line. Both visual acuity and OCT findings were stable at the three-month follow-up visit (Figure 3C). As the response to treatment was not complete at one month, the initial treatment was continued for three months when the medication was eventually tapered.

##  DISCUSSION

Diabetic macular edema is a major complication of diabetic retinopathy and one of the leading causes of visual impairment in the working-age population.^[1]^ Inflammation has an important role in the pathogenesis of diabetic retinopathy and DME. Systemic and local inflammatory biomarkers have been proven to have an important role in the development and progression of DME.^[15]^

Based on the importance of inflammatory processes in the pathogenesis of DME, systemic low-dose infliximab (TNF-α inhibitor) was employed in the treatment of the late-stage vision-threatening refractory DME with promising results.^[16]^ INF-α might be effective in the treatment of DME due to its opposite effects on TNF-α.^[12]^ This means that an increase in concentration of INF-α in a viable microenvironment can decrease the production and concentration of TNF-α and vice versa. Moreover, INF-α has both anti-inflammatory and anti-proliferative properties. It inhibits the production of VEGF in addition to IL-8 and TNF- α, two major local cytokines in the development of DME.^[8,9][10][11][12][14][15][16]^ The sub-tenon injection of INF-α2a was demonstrated to be effective in the treatment of refractory DME^[14]^; however, to the best of our knowledge, topical INF-α2b has not been previously employed in the treatment of DME.

Despite being a large molecule, its adequate penetration through the sclera and cornea has been demonstrated in previous studies.^[13,14,17]^ The safety of topical use of INF-α2b has also been shown in previous studies and no major systemic or local side effects have been reported in the treatment of patients with ocular surface tumors with the same dose.^[13]^

In the three patients in this series, all of the commonly used treatment modalities either failed due to ineffectiveness or were too expensive for the patients. These patients were finally started on topical INF-α2b four times a day with the anticipation that based on the importance of inflammatory processes in the development and progression of DME, this treatment would be effective. The treatment plan was continued for eight to twelve weeks based on the clinical judgment and OCT findings. The treatment dose was tapered by one drop every eight weeks based upon established ocular inflammatory disease therapies and our successful experience of employing topical INF-α2b in the treatment of refractory cystoid macular edema (CME).^[13]^

In these presented cases, topical INF-α2b was started when the effects of previous therapies such as intravitreal injections and MPC were proven futile and the patients needed further intervention. Comparison of OCT findings before and one-month after the treatment demonstrated an impressive improvement in macular structures and thickness in all cases. These findings were stable or improved at the three-month follow-up visit. Conjunctival injection and follicular conjunctivitis were the observed side effects and were treated with lubricants and low potency steroids.

In conclusion, this case series demonstrates the effectiveness of topical interferon-α2b in the treatment of selected cases of refractory DME. This therapy might also be an option in selected patients who develop complications after intravitreal injections or in patients who have contraindications to intravitreal injections. However, more robust studies such as randomized clinical trials are required to support our findings.

##  Financial Support and Sponsorship

Nil.

##  Conflicts of Interest

There are no conflicts of interest.
